# Review: Peering through a keyhole: liquid biopsy in primary and metastatic central nervous system tumours

**DOI:** 10.1111/nan.12553

**Published:** 2019-05-14

**Authors:** L. Bertero, G. Siravegna, R. Rudà, R. Soffietti, A. Bardelli, P. Cassoni

**Affiliations:** ^1^ Pathology Unit Department of Medical Sciences University of Turin Torino Italy; ^2^ Pathology Unit Città della Salute e della Scienza University Hospital, Turin Torino Italy; ^3^ Department of Oncology University of Turin Candiolo (Turin) Italy; ^4^ Candiolo Cancer Institute FPO‐IRCCS Candiolo (Turin) Italy; ^5^ Neuro‐oncology Unit Department of Neurosciences University of Turin Italy; ^6^ Neuro‐oncology Unit Città della Salute e della Scienza University Hospital Turin Italy

**Keywords:** cfDNA, circulating tumour cells, CNS tumours, CSF, liquid biopsy, molecular profiling

## Abstract

Tumour molecular profiling by liquid biopsy is being investigated for a wide range of research and clinical purposes. The possibility of repeatedly interrogating the tumour profile using minimally invasive procedures is helping to understand spatial and temporal tumour heterogeneity, and to shed a light on mechanisms of resistance to targeted therapies. Moreover, this approach has been already implemented in clinical practice to address specific decisions regarding patients’ follow‐up and therapeutic management. For central nervous system (CNS) tumours, molecular profiling is particularly relevant for the proper characterization of primary neoplasms, while CNS metastases can significantly diverge from primary disease or extra‐CNS metastases, thus compelling a dedicated assessment. Based on these considerations, effective liquid biopsy tools for CNS tumours are highly warranted and a significant amount of data have been accrued over the last few years. These results have shown that liquid biopsy can provide clinically meaningful information about both primary and metastatic CNS tumours, but specific considerations must be taken into account, for example, when choosing the source of liquid biopsy. Nevertheless, this approach is especially attractive for CNS tumours, as repeated tumour sampling is not feasible. The aim of our review was to thoroughly report the state‐of‐the‐art regarding the opportunities and challenges posed by liquid biopsy in both primary and secondary CNS tumours.

## Introduction

The possibility of detecting circulating tumour biomarkers in body fluids, including cerebrospinal fluid (CSF), has been exploited in routine clinical practice for tumour diagnosis, treatment and follow up for a long time 1, 2, 3. Protein markers are commonly used, but this approach does not provide information regarding the tumour's molecular profile 3. Comprehensive molecular profiling of a neoplasm has now been made possible by the analysis of circulating tumour components, and specific applications of this approach, which are usually referred to as ‘liquid biopsy’, are exponentially growing for both research and patient care 4.

Central nervous system (CNS) tumours are an unmet need: outcome is often dismal, and therapies offer limited efficacy. Tumour molecular profiling is especially relevant in this setting, but the CNS is a peculiar anatomic/functional compartment and even a biopsy can carry significant risks. Thus, liquid biopsy could fulfil its potential in this setting, although CNS specificities apply to this tool too. For example, the blood brain barrier (BBB) hinders the circulation of tumour components from the CSF to the blood, thus potentially impairing CNS tumour sampling through the commonly used blood‐based approaches.

Over the last few years, a significant amount of data have been accrued regarding liquid biopsy in both primary and secondary CNS tumours. The aim of our review is to thoroughly report the state‐of‐the‐art regarding the opportunities and challenges posed by liquid biopsy in this setting.

## The role of blood brain barrier

Although blood is an ideal, easily accessible source for liquid biopsy sampling and is routinely used to identify many tumour types, data show that its sensitivity can be severely limited when dealing with CNS neoplasms. The main factor hampering the detection of CNS tumour components in blood is the BBB. The latter is a peculiar anatomic and functional structure aimed at regulating the traffic of molecules and cells into and outside of the CNS. Its functions rely on the synergic activity of a wide range of partners including endothelial cells, pericytes and astrocytes 5. The widespread presence of tight junctions allows the passage of very small molecules only (less than a few nanometres) and this could explain the overall poor sensitivity observed by blood sampling when looking for components derived from CNS neoplasms 6.

However, as will be discussed, in specific situations it seems to be possible to detect the molecular signatures of CNS tumours in blood. For example, larger tumour volumes and the presence of contrast enhancement were correlated with an increased sensitivity for *IDH1* mutations 7. This result can be understood if we consider that contrast enhancement is a sign of BBB leakage, which is usually associated with higher grade tumours.

**Figure 1:**
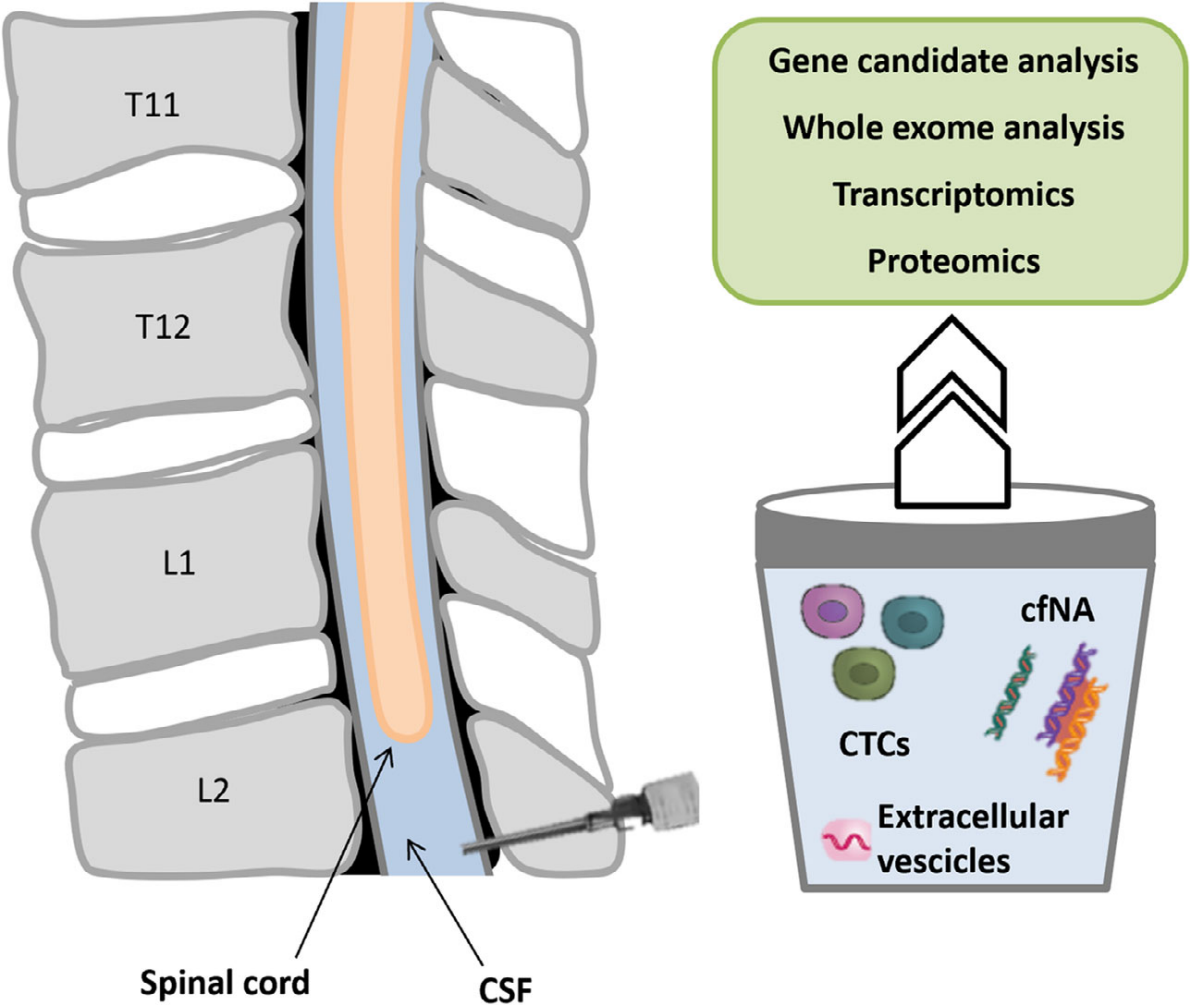
Cerebrospinal fluid (CSF) sampling for liquid biopsy. CSF, usually sampled by lumbar puncture, allows gathering of multiple tumour components which can be submitted to a wide range of molecular tests.

To overcome this limitation, CSF seems the best alternative source for sampling of CNS neoplasms considering its direct contact with CNS structures. Present data suggest a higher sensitivity of CSF compared with blood (Figure 1). Nevertheless, CSF sampling is not free of risks especially for patients with CNS neoplasms, although it is usually a feasible and safe approach.

A different strategy could be to alter the BBB permeability to allow the transfer of tumour‐derived markers into the blood. Until recently, this research topic was mostly aimed at increasing drug penetration into the CNS 8, but it is now being investigated as a tool to increase the sensitivity of liquid biopsy for CNS tumours; for example, focused ultrasound enabled blood‐based liquid biopsy in animal models of glioblastoma 9. The specific mechanisms through which the BBB hampers the transfer of specific tumour components deserve further study.

Last, it should be remembered that the BBB plays an active role in facilitating or preventing brain metastases (BM) from systemic tumours. Primary tumour cells can impair the BBB by releasing specific molecules such as nucleic acids or proteins, thus increasing the risk of BM development. Improved understanding of these mechanisms could pave the way not only to early detection of BM, but could also allow BM risk prediction or prevention 10.

## Technical considerations: what to analyse and how

**Figure 2:**
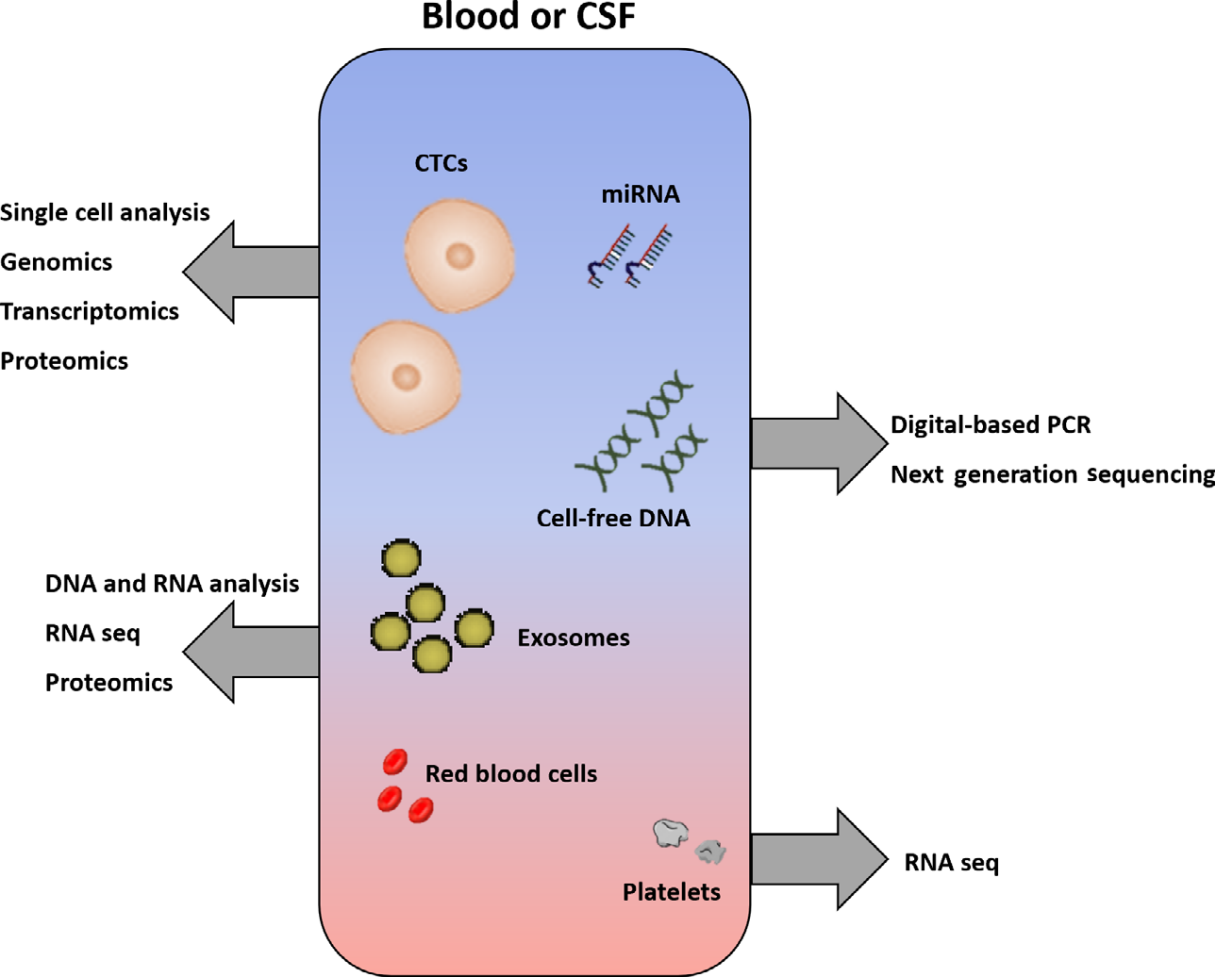
Liquid biopsy analytes and relevant assays. Different tumour components can be collected in liquid biopsy sources (blood or CSF) allowing a wide range of analyses.

The number of potential technical approaches is both extremely high and rapidly evolving 4. The first choice is about which tumour component or marker would be the most informative in the specific clinical setting (Figure 2). Analysis of circulating cell‐free nucleic acids (cfNA), including cfDNA and cfRNA, is the most used approach for the time being, potentially allowing the detection of somatic mutations, insertions, deletions, copy number variations and also enabling assessment of methylation status and of regulatory nucleic acids, such as microRNAs (miRNA) or long noncoding RNAs (lncRNA). These data are what usually matters the most to the pathologist and the clinician to achieve diagnosis and molecular profiling for clinical management. Conversely, cfNA analysis does not allow reliable assessment of RNA expression, but this limitation can be overcome by analysing circulating tumour cells (CTCs) or extracellular vesicles (EVs) which also allow proteomic studies 11, 12.

Another important choice regarding genomic studies is whether to use a candidate gene strategy or larger‐scale approaches like whole exome sequencing. In the diagnostic clinical setting, the first is usually advised: (i) it allows reliable assessment of a specific set of genes with known diagnostic and/or predictive value; (ii) it enables very low limits of detection; (iii) it provides a faster, clinically suitable, turnaround time with lower costs; (iv) bioinformatic support is usually not required; (v) germline DNA analysis and its ethical/legal implications can usually be avoided. Conversely, for exploratory/research studies, extensive approaches, also including deep sequencing, can be considered. Among candidate gene analysis techniques, droplet digital PCR allows an extremely low limit of detection (0.001%) and provides precise DNA copy number quantification 13, thus allowing monitoring of residual disease.

For CTCs and EVs analysis, different strategies have to be used for primary and secondary CNS tumours as these tumour components have to be selectively captured and enriched; however, the proposed approaches will be discussed in the relevant paragraphs. A further informative component worthy of mention is circulating platelets. Analysis of tumour‐educated platelets by RNA sequencing has been proven to be highly informative in both primary and secondary CNS neoplasms 14, 15.

## Liquid biopsy in primary CNS tumours

Molecular profiling is of paramount importance for the characterization of primary CNS tumours. For this reason, the latest WHO classification of these tumours introduced the concept of ‘integrated diagnosis’: a diagnosis based upon the integration of both morphological and molecular findings. This approach is necessary to distinguish between entities with significant biological and clinical differences, but overlapping histological features 16.

Even considering just the group of diffuse gliomas, a wide range of molecular alterations is relevant for their proper classification and treatment: *IDH1*/*2*,* ATRX*,* TP53*,* TERT* promoter and histone H3‐coding genes mutations, 1p/19q chromosomal arms codeletion, *EGFR* alterations and *MGMT* promoter methylation 16, 17. Overall, specific molecular hallmarks are relevant for diagnosis of most primary CNS tumours, while assessment of temporal molecular heterogeneity could help shed a light on mechanisms of resistance to treatments. Thus, liquid biopsy could be extremely useful for the diagnosis and follow up of primary CNS tumours.

## Circulating cell‐free nucleic acids analysis

Analysis of the circulating cfNA shed by tumour cells allows detection of gene alterations, including mutations or fusions, changes in methylation profile, copy number variations and to quantify tumour burden; moreover, overall cfDNA (i.e. tumour and non‐tumour derived) concentration has been suggested to play a prognostic role in primary CNS tumours 18.

Boisselier *et al*. evaluated the detection of *IDH1* mutations in plasma cfDNA of patients with histologically proven glioma with known tumour IDH‐status:^7^ sensitivity and specificity were 60% and 100%, respectively (Table 1). Sensitivity correlated with tumour volume and contrast enhancement: a higher contrast‐enhancing tumour volume was associated with higher sensitivity. More recently, Bettegowda *et al*. investigated the possibility of detecting a wide range of alterations in a large series of tumour types by digital PCR analysis of plasma cfDNA 19. A cohort of 41 primary brain tumours (including gliomas and medulloblastomas) was included and, unlike most extra‐CNS tumours, the detection rates were limited (<50% for medulloblastoma and <10% for gliomas).

**Table 1:** Studies investigating circulating cfDNA in primary CNS tumours

	Tumour	Positive CSF cytology	Positive CSF molecular profiling	Positive blood molecular profiling
Rhodes *et al*. 1	Glioblastoma	ND/NR	1/1 (100%)	ND/NR
Rhodes *et al*. 2	Glioblastoma	ND/NR	1/1 (100%)	ND/NR
Boisselier *et al*. 7	Glioma (low grade = 8, high grade = 17)	ND/NR	ND/NR	Low grade: 3/8 (37.5%) High grade: 12/17 (70.6%)
Salkeni *et al*. 30	Glioblastoma	ND/NR	ND/NR	3/3 (100%)
Bettegowda *et al*. 19	Glioma (*n* = 27) Medulloblastoma (*n* = 14)	ND/NR	ND/NR	Glioma: <10% Medulloblastoma: <50%
Pan *et al*. 36	Meningioma (*n* = 1) Schwannoma (*n* = 1)	ND/NR	Meningioma: 1/1 (100%) Schwannoma: 0/1 (0%)	Meningioma: 0/1 (0%) Schwannoma: 0/1 (0%)
De Mattos‐Arruda *et al*. 20	Glioblastoma (*n* = 4) Medulloblastoma (*n* = 2)	ND/NR	Glioblastoma: 4/4 (100%) Medulloblastoma: 2/2 (100%)	Glioblastoma: 0/4 (0%) Medulloblastoma: 0/2 (0%)
Wang *et al*. 22	Low‐grade glioma (*n* = 8) High‐grade glioma (*n* = 13) Ependymoma (*n* = 7) Medulloblastoma (*n* = 6) Other low‐grade tumour (*n* = 1)	ND/NR	Low‐grade glioma: 6/8 (75%) High‐grade glioma: 13/13 (100%) Ependymoma: 5/7 (71%) Medulloblastoma: 5/6 (83%) Other low‐grade tumour: 1/1 (100%)	ND/NR
Pentsova *et al*. 21	Glioma (*n* = 8) Ependymoma (*n* = 1)	Glioma: 0/8 Ependymoma: 0/1	Glioma: 6/8 (75%) Ependymoma: 0/1 (0%)	ND/NR
Connolly *et al*. 23	Ependymoma (*n* = 3)	ND/NR	0/3 (0%)	0/3 (0%)
Huang *et al*. 32	Diffuse midline glioma (*n* = 5)	ND/NR	4/5 (80%)	ND/NR
Martìnez‐Ricarte *et al*. 31	High‐grade glioma (*n* = 15) Low‐grade glioma (*n* = 5)	ND/NR	High‐grade glioma: 15/15 (100%) Low‐grade glioma: 2/5 (40%)	ND/NR
Pan *et al*. 33	Brainstem glioma	ND/NR	39/40 (98%)	3/8 (38%)
Panditharatna *et al*. 34	Diffuse midline glioma	ND/NR	24/27 (89%)	34/40 (85%)
Hiemcke‐Jiwa *et al*. 37	Lymphoplasmacytic lymphoma (*n* = 6) PCNSL (*n* = 1)	Lymphoplasmacytic lymphoma: 2/6 (33%) PCNSL: 1/1 (100%)	Lymphoplasmacytic lymphoma: 5/6 (83%) PCNSL: 1/1 (100%)	ND/NR
Miller *et al*. 24	Diffuse glioma (grade II‐III‐IV)	7/80 (9%) CSF cytology not available in five cases	42/85 (49%)	3/19 (16%)

CNS, central nervous system; CSF, cerebrospinal fluid; ND/NR, not done/not reported; PCNSL, primary central nervous system lymphoma.

*Notes:* (i) CSF molecular profiling was considered positive if at least one tumour mutation was detected in cfDNA, but in some cases only a subset of alterations was detected compared to tissue samples; (ii) Cases with negative/not available tissue profiling and negative liquid profiling were excluded. Conversely, cases with positive CSF despite negative or non‐evaluated tissue sample were considered; (iii) Atypical cells were considered positive in terms of CSF cytology evaluation.

The possibility of using CSF as a source for cfDNA analysis in primary CNS tumours was suggested more than 20 years ago by Rhodes *et al*. who identified *EGFR* amplification and a *TP53* mutation in glioblastoma patients (Table 1) 1, 2. De Mattos‐Arruda *et al*. further showed that CSF‐derived cfDNA analysis detects mutations of both primary and secondary brain tumours 20. Regarding the primary neoplasms, four glioblastomas and two medulloblastomas were investigated; at least one tumour mutation was detected in all cases by CSF cfDNA analysis, while blood‐derived liquid biopsies were negative. Tumour burden, assessed by cfDNA quantification, mirrored the neuroimaging findings, thus supporting liquid biopsy as an effective tool to track tumour evolution over time and possibly foresee recurrence/progression, although specific prospective studies are needed for clinical validation. Pentsova *et al*. confirmed the usefulness of CSF liquid biopsy, but observed a low sensitivity (6/9 patients, 66.7%) in a series of primary CNS tumours, mostly including grade III–IV (WHO) diffuse gliomas 21.

Tumour variables, such as histological type, grade and site, could influence the efficacy of cfDNA analysis. Medulloblastoma, for example, a malignant cerebellar tumour with high cellularity could be expected to more easily shed cfDNA into CSF compared to hemispheric diffuse low‐grade gliomas with an infiltrative growth pattern. This hypothesis is supported by Wang *et al*. who found that tumours closer to CSF spaces have a higher probability of being detected by CSF cfDNA analysis compared to tumours not abutting the ventricles 22. This finding was true for different tumour types, including high‐grade gliomas, ependymomas and medulloblastomas. Interestingly, Connolly *et al*. analysed CSF and blood samples of three cases of grade II intramedullary ependymomas without identifying any alteration in blood or in CSF 23. In this regard, other variables such as CSF flow alterations may affect cfDNA circulation and liquid biopsy sensitivity.

A very recent study specifically investigated a large series of diffuse gliomas (*n* = 85), including 46/85 (54%) glioblastomas and 39/85 (46%) lower grade gliomas (grade II–III), using a targeted next‐generation sequencing‐based assay 24. CSF was collected by lumbar puncture in most cases (82/85, 96%). Overall sensitivity was of 49.4% (42/85): 59% (27/46) in glioblastomas and 38% (15/39) in lower grade gliomas, although a significant association between tumour grade and CSF circulating tumour DNA (ctDNA) detection was not found. Conversely, the presence of ctDNA was associated with tumour progression, a higher tumour burden, tumour spread in the ventricular system/subarachnoid space and a shorter survival time since CSF collection. This latter finding was found to be independent of tumour burden. Regarding the diagnostic efficacy of this approach, in a subset of 10 lower grade gliomas with available tissue sample, the genetic alterations currently used to define the glioma subtype according to WHO criteria were always consistent between CSF and tissue. Plasma was analysed in 19 cases with positive CSF cfDNA and no mutations were detected in most of them (16/19, 84%), while in the remaining samples, the mutant allele frequencies were very low. An important finding regarding tumour heterogeneity was that, as the time between tissue and CSF sampling increased, a greater genetic divergence was observed, in particular for genes involved in growth factor signalling pathways. These data highlight the importance of longitudinal molecular evaluation in diffuse gliomas and show that liquid biopsy may successfully fulfil this need.

*EGFR* alterations are another relevant molecular hallmark as *EGFR* amplification and/or EGFRvIII variant are present in many glioblastomas 25, 26 and have been targeted in many clinical trials employing a wide range of strategies (i.e. direct inhibition, vaccination, antibody‐drug conjugate therapy). Unfortunately, the results have been unsatisfactory so far 27, but new strategies are currently under investigation 28, 29. Salkeni *et al*. were able to detect EGFRvIII in plasma‐derived cfDNA of 3/3 patients with EGFRvIII‐positive tumours (out of a cohort of 13 glioblastomas), making this marker potentially assessable through liquid biopsy 30.

Regarding the diagnostic potential of CSF cfDNA analysis, evaluation of a selected panel of molecular alterations in CSF cfDNA allowed the identification of tumour molecular alterations in 17/20 (85%) cases of diffuse gliomas, allowing their classification according to the latest WHO criteria 31. Also, detection of mutations affecting histone H3 genes in a case series of paediatric brain tumours showed high sensitivity and specificity: 87.5% and 100% respectively 32. This finding is especially relevant as the H3 K27M mutation characterizes, although not exclusively, a subgroup of highly malignant midline diffuse gliomas, usually occurring in children. Considering the location of these tumours, which often involve the brainstem, this approach could enable to achieve diagnosis without the potential risks of a surgical biopsy.

Recent data further support the sensitivity of liquid biopsy for brainstem gliomas: Pan *et al*. evaluated a series of 57 patients and were able to detect at least one tumour‐specific mutation in 82.5% (47/57) of cases 33. Sensitivity reached 96.3% (36/37) when considering only patients with a detectable mutation in the tumour tissue sample, while in 83% (31/37), it was possible to achieve the same results as tissue analysis by liquid biopsy. CSF and plasma cfDNA were compared in eight patients producing sensitivities of 100% and 37.5% respectively. Higher levels of cfDNA, tumour directly abutting the CSF spaces and higher tumour grade were associated with an effective liquid biopsy, while a trend was observed for higher tumour volumes. An important consideration is that in most cases (91.2%, 52/57), CSF was collected intraoperatively, thus, results could differ from lumbar puncture sampling. Last, in three cases out of 10 with negative tissue analysis, mutations were detected in cfDNA. This result could support the capability of liquid biopsy to recapitulate tumour heterogeneity, but it must be interpreted cautiously as sequencing depths differed significantly between the two assays. Another recent study by Panditharatna *et al*. focused on midline gliomas and evaluated H3 K27M mutations and typical partner alterations in cfDNA by droplet digital PCR 34. CSF analysis sensitivity reached 87% (20/23) in H3 K27M‐mutant cases (defined through tissue analysis), but, interestingly, plasma sensitivity was very high, ranging from 90% (18/20) in the original series to 80% (16/20) in a prospective cohort collected during a clinical trial. However, mutant allele fractions were lower compared to CSF. The relationship between sampling site and mutations detection was also evaluated: a higher mutant allele frequency was observed if CSF was collected adjacent to the tumour location, but mutant alleles could be potentially detected in all sample sites. Notably, longitudinal comparisons showed a correlation between clinical course, radiological findings and mutant allele frequencies.

A concluding remark regarding the use of liquid biopsy as a diagnostic tool for gliomas is that particular care should be applied when selecting the gene panel to be assessed, as dramatic differences exist between the molecular landscapes of adult and paediatric tumours. Moreover, reliance on single markers should be avoided. For instance, the H3 K27M mutation, which was initially considered as a potential pathognomonic alteration of malignant diffuse brainstem glioma, has been reported in a wider range of tumours 35.

Liquid biopsy could be useful for the diagnosis and follow up of non‐glial primary brain tumours as well, although less data are available 36. Hiemcke‐Jiwa *et al*. demonstrated the possibility of detecting *MYD88* mutations, a common alteration of primary central nervous system lymphomas (PCNSL) (Table 1) 37. This is especially intriguing considered that PCNSL are usually extremely chemo/radiosensitive, thus surgery could be avoided.

Epigenetic alterations are also relevant biomarkers in primary brain tumours. The possibility of assessing *MGMT* promoter methylation in blood‐derived cfDNA was first reported by Weaver *et al*. in a small case series of high‐grade diffuse gliomas, achieving a 50% sensitivity 38. Lavon *et al*. and further studies confirmed this possibility and also explored the correlations with outcome and longitudinal evolution during disease course 39, 40, 41. cfDNA methylation analysis has also been proposed for diagnostic purposes in gliomas using *CDKN2A* promoter methylation assessment 42.

Analysis of circulating micro RNAs (miRNAs) is another potential strategy. miRNAs are noncoding short RNA regulatory molecules, which can widely affect transcription and translation. Most available data relate to diffuse glioma, in particular glioblastoma, and a broad range of miRNAs have been investigated, alone or in specific combinations, as possible diagnostic or prognostic biomarkers 43, 44, 45, 46, 47, 48, 49, 50, 51, 52. Both CSF 44, 45, 49, 51 and blood [43, 46, 47, 48, 50, 52] have been evaluated as sources of genetic material. The possibility of distinguishing between primary and secondary CNS tumours by miRNA profiling, and of estimating patients’ outcome based on their concentrations has been suggested.

Overall, the main limitation of this approach is the heterogeneity of the reported data. Although many different candidate miRNAs have been proposed, their validation in larger cohorts is lacking, hampering translation into clinical practice.

Last, the possibility of exploiting other nucleic acids such as long noncoding RNAs or mitochondrial DNA should also be taken into consideration 53, 54.

## Circulating tumour cells

Circulating tumour cells (CTCs) analysis is another potential approach for liquid biopsy and allows the assessment of nucleic acids, including mRNAs, which are usually quickly degraded in the circulation, and other tumour cell components such as cytoplasmic proteins. Detection and capture of CTCs are challenging due to their extremely low concentration in blood, thus many different techniques have been investigated 55. Moreover, primary CNS tumour cells do not express a common membrane marker, thus hampering their detection and collection.

The presence of blood CTCs in primary CNS tumours was debated due to the extremely low incidence of extra‐CNS metastases 56. However, blood CTCs were successfully detected in a series of 33 glioblastomas using a combination of markers (SOX2, Tubulin beta‐3, EGFR, A2B5 and c‐MET) with a sensitivity (defined as the rate of patients with at least one positive sample) of 13/33 (39%) 57. Specific probes for molecular hallmarks of glioblastoma, such as telomerase activity, have been also investigated. MacArthur *et al*. observed a sensitivity of 8/11 (72%) in a series of grade III and IV gliomas sampled before radiotherapy 58. Interestingly, sampling after radiotherapy was successful in only 1/8 (12.5%). Muller *et al*. were able to detect circulating neoplastic cells in 21% (29/141) of patients with glioblastoma by GFAP immunostaining of mononuclear circulating cells 59. Moreover, an integrated positive and negative selection approach was also effective in grade II diffuse gliomas reaching a sensitivity of 82% (9/11) in this specific subset of tumours. Using this assay, CTCs count resulted associated with tumour status (i.e. progression *vs*. radiation necrosis) in an additional series of five cases 60. The significant variability in terms of detection rates is likely due to the differences in technical approaches and to the overall low sample sizes. Further, larger studies are warranted to confirm the real efficacy of the different analytical methods.

Last, an interesting observation regarding tumour heterogeneity was reported by Sullivan *et al*.: CTCs can show a different gene expression profile compared to the previously resected tumour sample. Specifically, increased mesenchymal differentiation and a reduced neural‐like profile were observed. These data suggest that a single approach or a single set of markers may be unable to fully sample tumour heterogeneity 57.

## Tumour‐educated platelets

Analysis of circulating platelets is another intriguing approach to achieve liquid biopsy of CNS tumours 61, 62. Many biomolecules are transferred between tumours and platelets and this process has been termed platelet “education” 63, 64. As initially shown by Nilsson *et al*., platelets can sequester tumour‐shed extracellular vesicles which allow detecting of tumour‐derived biomarkers, including proteins and nucleic acids like the EGFRvIII mRNA, with high sensitivity and specificity 14. Different patterns of mRNA expression were also observed in platelets of patients with glioblastoma compared with healthy controls. The shaping of platelets mRNA splicing by the external environment can thus be exploited for tumour diagnostics and profiling. Indeed, Best *et al*., showed that analysis of RNA splicing profiles of tumour‐educated platelets was able to identify patients with tumours and further distinguish those with localized or metastatic disease. In particular, this approach correctly classified 85% (33/39) of individuals as patients with a neoplasm in a series of 39 glioblastomas. Regarding the specific diagnostic potential, 78% of glioblastoma patients were correctly classified when compared with controls and five other tumour types in a training series of 175 samples. Similar results were observed in a validation set of 108 cases. Furthermore, in a series of 114 samples (62 patients) with brain lesions, tumour‐educated platelet analysis allowed correct identification of cancer patients in 91%, discrimination between primary and secondary tumours in 93% and discerned between the mutational subtypes in 82% 15. It has also to be remembered that blood components can actively promote glioma growth and malignancy, thus further studies investigating strategies to antagonize this cross‐talk are warranted 65.

## Extracellular vesicles and other approaches

Extracellular vesicles (EVs) are a heterogeneous range of membrane‐bound carriers secreted or shed by both normal and neoplastic cells. Many studies have investigated their physiological role in intercellular communication, which can occur between different tissues or compartments 66.

Similar to CTC, liquid biopsy by EVs analysis offers some advantages over cfNAs, allowing mRNAs characterization and evaluation of cytoplasmic proteins 67. In primary brain tumours, this approach has been mainly investigated in malignant gliomas, and both blood and CSF have been explored as potential sources of EVs. As initially shown by Skog *et al*. 11, EVs shed by glioblastoma contain a wide range of molecules, including nucleic acids and proteins, and can significantly affect distant cells, for instance by increasing proliferation or angiogenesis. Moreover, specific molecular hallmarks of gliomas can be detected in EVs: for example, Skog *et al*. were able to detect EGFRvIII in EVs of 7/25 (28%) patients with glioblastoma 11.

More recently, Chen *et al*. detected *IDH1* mutations in CSF‐derived EVs of patients with IDH‐mutant gliomas using highly sensitive techniques; conversely, serum‐derived EVs of the same patients were negative 68. Although based on significantly different approaches and techniques, these results somehow conflict with data reported by Boisselier *et al*. 7 and indicate the importance of further research evaluating how the interplay between biological and technical variabilities can affect the results. *EGFR* alterations can also be assessed in EVs. 11, 69

Concerning immune system evasion, a recent study identified PD‐L1 in glioblastoma‐derived EVs and the presence of this ligand was associated with impaired T‐cell activation 70.

miRNAs are another cargo of EVs through which they can exert their regulatory functions on distant tissues 71, 72, 73. As for circulating free miRNAs, many candidates have been investigated as potential diagnostic biomarkers and different subsets of EVs have been compared 74, 75. A panel of three miRNAs (miR‐21, miR‐222 and miR‐124‐3p), detected in blood‐derived exosomes, was recently shown to be differentially expressed in high‐grade gliomas compared with low‐grade gliomas or healthy controls 76.

Last, circulating proteins and metabolites can be also exploited as biomarkers of neoplastic diseases, including brain tumours. The improved knowledge of tumour molecular profiles and their correlation with cellular metabolism could enable new avenues for diagnosis and monitoring of CNS neoplasms, as recently shown by Ballester *et al* 77. This extensive topic, however, is outside the scope of the present review.

## Liquid biopsy in secondary CNS tumours

Brain metastases are the most frequent intracranial tumours. They develop in 9–17% of patients with solid neoplasms (usually from lung and breast cancers and melanoma) and their incidence is increasing 78, 79. Quality of life and outcome are severely affected by BMs 80, but encouraging results have been recently achieved, thanks to targeted therapies and immunotherapeutic drugs 81.

Brain metastases from solid tumours often show a divergent molecular profile compared to the primary lesion. Spatial and temporal heterogeneity is an intrinsic characteristic of malignant tumours and this hallmark is strictly intertwined with the continuous evolutionary pressure which selects the neoplastic cells that best fit in a specific anatomic compartment or timepoint during the course of disease, also because of external variables, such as ongoing treatments. Brastianos *et al*., showed that more than 50% of BMs harbour private molecular alterations (compared to the primary tumour or extra‐CNS lesions) which are potentially actionable 82. These data suggest that, to offer the optimal treatment to these patients, BM sampling is necessary to fully recapitulate tumour heterogeneity; as surgical resection of BMs is not indicated in most cases, liquid biopsy could help overcome this limitation.

Neoplastic meningitis (NM) is a specific type of secondary CNS involvement which is characterized by the spread of neoplastic cells across the leptomeninges and their circulation through the CSF. Definitive diagnosis is based upon cytological demonstration of malignant cells in the CSF, but sensitivity is limited and thus diagnosis usually relies on supporting clinical and neuroimaging findings 83. Liquid biopsy could be a valuable diagnostic/profiling tool also in this setting.

## Circulating cell‐free nucleic acids analysis

The possibility of detecting tumour‐derived cfDNA in patients with secondary CNS involvement has long been reported 1, 84, but only during the last few years this approach has received specific consideration as a potential tool for the routine care of patients 20, 21, 36, 85, 86, 87, 88, 89, 90, 91, 92, 93, 94, 95, 96, 97. Most data have been obtained in NM from non‐small‐cell lung carcinoma (NSCLC), but studies on breast carcinoma 1, melanoma 90, 98 and secondary haematological malignancies 97 have also been reported (Table 2).

**Table 2:** Studies investigating circulating cfDNA in secondary CNS tumours from solid neoplasms

	Primary tumour	Type of CNS involvement	Positive CSF cytology	Positive CSF molecular profiling	Positive blood molecular profiling
Rhodes *et al*. 1	Breast	NM	ND/NR	1/1 (100%)	ND/NR
Swinkels *et al*. 84	NSCLC	NM	0/2 (0%)	2/2 (100%)	ND/NR
Shingyoji *et al*. 85	NSCLC	NM	10/21 (47.6%)	13/21 (61.9%)	ND/NR
Yang *et al*. 88	NSCLC	BM and NM	ND/NR	BM: 2/5 (40%) NM: 4/4 (100%)	ND/NR
De Mattos‐Arruda *et al*. 20	Breast and lung	BM and NM	BM: 13/24 (54.2%) NM: 1/3 (33%)	BM: 17/17 (100%) NM: 3/3 (100%)	0% in CNS‐restricted disease
Pan *et al*. 36	Mixed	BM and NM	BM: ND/NR NM: 2/2 (100%)	BM: 5/5 (100%) NM: 1/1 (total DNA was analysed)	BM: 3/5, 2/3 with also extra‐CNS progression NM: ND/NR
Sasaki *et al*. 89	NSCLC	NM	2/7 (28.6%)	7/7 (100%)	0/3 (0%)
Li *et al*. 90	Melanoma	NM	1/1 (100%)	1/1 (100%)	0/1 (0%)
Pentsova *et al*. 21	Mixed	BM and NM	BM: ND/NR NM: 3/3 (100%)	BM: 17/24 (70.8%) NM: 3/3 (100%)	ND/NR
Zhao *et al*. 96	NSCLC	NM		7/7 (100%)	2/7 (28.6%), 1/2 with also extra‐CNS progression
Marchiò *et al*. 91	NSCLC	NM	2/2 (100%)	2/2 (100%)	0/2 (0%)
Siravegna *et al*. 93	Breast	NM	ND/NR	1/1 (100%)	1/1 (100%), but showed decreasing mutant allele frequencies despite CNS disease progression
Huang *et al*. 94	CUP	NM	1/1 (100%)	1/1 (100%)	ND/NR
Li *et al*. 95	NSCLC	NM	18/28 (64%)	28/28 (100%)	19/26 (73.1%)
Ballester *et al*. 77	Melanoma	NM	3/3 (100%)	2/3 (67%)	ND/NR
Nanjo *et al*. 100	NSCLC	NM	5/13 (38%)	5/13 (38%)	ND/NR

BM, brain metastasis; CNS, central nervous system; CSF, cerebrospinal fluid; CUP, cancer of unknown primary; NM, neoplastic meningitis; NSCLC, non‐small cell lung cancer; ND/NR, not done/not reported.

*Notes:* (i) CSF molecular profiling was considered positive if at least one tumour mutation was detected in cfDNA, but in some cases only a subset of alterations was detected compared to tissue samples; (ii) In many cases, NM was present together with BM: these cases were considered together with NM only cases; (iii) Cases with negative/not available tissue profiling and negative liquid profiling were excluded. Conversely, cases with positive CSF despite negative or non‐evaluated tissue sample were considered; (iv) Atypical cells were considered positive in terms of CSF cytology evaluation.

cfDNA analysis proved a quite high overall sensitivity as a diagnostic tool for BMs or NM from solid tumours, ranging from 40% to 100%, but sample sizes of the available studies are still limited. Nevertheless, if we compare this tool with traditional cytology (the gold‐standard for NM diagnosis at present) 99, cfDNA appears to be superior (Table 2). Another intrinsic advantage of liquid biopsy compared with cytology is that it allows tumour molecular profiling, while cytology can only provide a diagnostic confirmation.

With respect to the variables potentially affecting liquid biopsy yield, the type of CNS involvement should be considered. BMs may have limited contact with CSF, especially when dealing with a single or few lesions in a supratentorial intraparenchymal location. Conversely, tumour cells circulation in the CSF is a hallmark of NM, and thus a higher sensitivity can be expected in the latter condition. The results of Yang *et al*. 88 and Pentsova *et al*. 21 support these considerations, although De Mattos‐Arruda *et al*. 20. and Pan *et al*. 36 were able to identify tumour cfDNA in all BMs cases (Table 2).

As for primary CNS tumours, the most pressing question is choice of the source for cfNAs (or of other analytes). Blood or other easily accessible body fluids (like saliva or urine) would obviously be preferable, but they may not be representative of CNS‐restricted lesions (Table 2). Some important considerations stem from the available data: i) a negative result may be obtained from blood‐based liquid biopsy even when active disease is present within the CNS and this is an important concern when dealing with minimal residual disease monitoring; ii) even if we find alterations in blood‐based liquid biopsy, these could be unrepresentative of the CNS disease.

Within the possible practical applications of liquid biopsy in this setting, CSF testing in patients with CNS involvement by NSCLC seems one of the most promising, mirroring the already widespread practice of blood‐based testing to promptly detect emerging resistance‐associated mutations in extra‐CNS NSCLC. As recently shown by Nanjo *et al*., the specific detection of the *EGFR* T790M mutation in CSF cfDNA was associated with clinical efficacy of treatment with osimertinib, a third‐generation EGFR tyrosine kinase inhibitor developed to overcome T790M‐induced resistance 100. Conversely, in patients with positive CSF liquid biopsy, but without *EGFR* T790M detection, no response to treatment was observed.

Last, it is worth pointing out that the significant differences observed between studies may depend on the wide range of techniques that have been tested so far. This variability should be addressed in future studies.

## Circulating tumour cells and other approaches

The presence of common membrane markers, like EpCAM, significantly helps CTCs collection in secondary CNS tumours, but alternative strategies are also possible 101. As a diagnostic tool, CSF CTCs proved to be superior to traditional cytology 102; moreover, the possibility of using this approach also for molecular profiling has been demonstrated. Magbanua *et al*. 86, 87 and Jiang *et al*. 92 were able to assess copy number and mutational profile of CSF CTCs in metastatic breast and lung cancer respectively. Similar to cfNAs analysis, CTCs collection by blood‐derived liquid biopsy was not possible or showed a low yield in cases with CNS‐restricted disease.

Best *et al*. showed that analysis of tumour‐educated platelets in patients with BMs allows identification of primary tumour type in 70% of samples 15. Moreover, blood component indices (like white blood cells or platelets values and ratios) may harbour prognostic significance in patients with BMs 103: this finding can be expected considered the role played by the systemic immune system in BM development 104.

Finally, EVs analysis in secondary CNS tumours is especially intriguing not only as a diagnostic/profiling tool, but also as a prognostic assay. Multiple studies suggested that tumour‐derived EVs may play an active role in creating a pro‐metastatic niche in distant tissues 105, including the CNS 10, 106. Thus, EVs characterization could allow to estimate the metastatic potential of a tumour and, possibly, to devise the therapeutic strategies capable of hampering this process even before BMs are established.

## Future perspectives

The recent technological advancements paired with the growing importance of molecular profiling in CNS neoplasms can explain the far‐reaching results achieved in just the last few years regarding liquid biopsy use in CNS tumours 107, 108.

The ever‐increasing reliance on molecular traits for proper classification of primary CNS tumours will favour the clinical adoption of liquid biopsy as a routine diagnostic tool in selected cases, for example, when surgical resection is not indicated. Conversely, resection and histological examination will probably remain the cornerstone diagnostic approach if feasible, considered its therapeutic relevance and the risk of diagnostic pitfalls due to overlapping molecular features even between significantly different tumour entities [for example, a pilocytic astrocytoma (grade I) can rarely harbour the H3 K27M mutation which is characteristic of diffuse midline gliomas (grade IV)]. Moreover, a tissue sample is needed for whole genome or proteomic studies which are now increasingly warranted in translational/clinical research protocols.

Liquid biopsy could instead represent a game‐changing development for disease follow up considering that repeated surgical sampling is not feasible in CNS tumours. Its first implementation, in this setting, could probably be as a companion tool for disease monitoring and tumour burden quantification: liquid biopsy data could help resolve conflicting clinical and radiological findings, for instance, when dealing with a suspected pseudo‐progression. The following step could be the longitudinal assessment of tumour heterogeneity: for example, patients with IDH‐mutant diffuse gliomas can have very‐long disease courses characterized by a progressive increase of tumour malignancy. Liquid biopsy could enable prompt detection and molecular characterization of disease progression, allowing optimization of clinical management. Nevertheless, the relevance of liquid biopsy in this setting will ultimately depend on the identification of significant, actionable, prognostic and/or predictive markers.

The same considerations apply to secondary neoplasms, but special caution should be applied when dealing with synchronous intra‐ and extra‐CNS disease progression, as blood‐derived results could be uninformative of the CNS disease. Nevertheless, the outcome of patients with BMs has dramatically changed in the last few years, thanks to the newly available targeted treatments 109, thus liquid biopsy is expected to become a mandatory assessment for an increasing number of tumour types and disease settings to optimize treatment decisions and prompt detection of resistance‐associated mutations.

## Conclusion

In the coming years, liquid biopsy will probably become a common tool for the diagnosis and follow up of both primary and secondary CNS tumours. For research purposes, liquid biopsy will be a cornerstone to determine longitudinal changes in the molecular profile of tumours, thus improving our knowledge of tumour resistance/progression mechanisms. Further studies, evaluating larger, prospective series, are needed to evaluate technical variabilities, validate its use in specific disease settings and, most importantly, to assess which is the ultimate clinical benefit for patients with CNS tumours.

## Funding information

This work was supported by the Rete Oncologica del Piemonte e della Valle d'Aosta (no specific grant number applicable) and received funding specifically dedicated to the Department of Medical Sciences from Italian Ministry for Education, University and Research (Ministero dell'Istruzione, dell'Università e della Ricerca ‐ MIUR) under the programme ‘Dipartimenti di Eccellenza 2018–2022’. Project n° D15D18000410001. G.S. was supported by a 3‐year FIRC‐AIRC fellowship and by ‘Roche per la ricerca’ ‐ Grant 2017. A.B. was supported by IMI contract n. 115749 CANCER‐ID.

## Disclosure

The authors declare they have no conflicts of interest.
